# Antihypertensive Medications for Severe Hypertension in Pregnancy: A Systematic Review and Meta-Analysis

**DOI:** 10.3390/healthcare10020325

**Published:** 2022-02-09

**Authors:** Adila Awaludin, Cherry Rahayu, Nur Aizati Athirah Daud, Neily Zakiyah

**Affiliations:** 1 Department of Pharmacology and Clinical Pharmacy, Faculty of Pharmacy, Universitas Padjadjaran, Bandung 40132, Indonesia; adila.awaludin6@gmail.com; 2 Department of Pharmacy, Dr. Hasan Sadikin General Hospital, Bandung 40161, Indonesia; cherryrahayu@gmail.com; 3 Discipline of Clinical Pharmacy, School of Pharmaceutical Sciences, Universiti Sains Malaysia, George Town 11800, Malaysia; aizati@usm.my; 4 Center of Excellence in Higher Education for Pharmaceutical Care Innovation, Universitas Padjadjaran, Bandung 40132, Indonesia

**Keywords:** high blood pressure, hypertension therapy, hypertension-induced pregnancy, severe preeclampsia

## Abstract

Background: Hypertension in pregnancy causes significant maternal and fetal mortality and morbidity. A comprehensive assessment of the effectiveness of antihypertensive drugs for severe hypertension during pregnancy is needed to make informed decisions in clinical practice. This systematic review aimed to assess the efficacy and safety of antihypertensive drugs in severe hypertension during pregnancy. Methods: A systematic review using the electronic databases MEDLINE (PubMed) and Cochrane Library was performed until August 2021. The risk-of-bias 2 tool was used to assess the risk-of-bias in each study included. Meta-analysis was conducted to assess heterogeneity and to estimate the pooled effects size. Results: Seventeen studies fulfilled the inclusion criteria and 11 were included in the meta-analysis. Nifedipine was estimated to have a low risk in persistent hypertension compared to hydralazine (RR 0.40, 95% CI 0.23–0.71) and labetalol (RR 0.71, 95% CI 0.52–0.97). Dihydralazine was associated with a lower risk of persistent hypertension than ketanserin (RR 5.26, 95% CI 2.01–13.76). No difference was found in the risk of maternal hypotension, maternal and fetal outcomes, and adverse effects between antihypertensive drugs, except for dihydralazine, which was associated with more adverse effects than ketanserin. Conclusions: Several drugs can be used to treat severe hypertension in pregnancy, including oral/sublingual nifedipine, IV/oral labetalol, oral methyldopa, IV hydralazine, IV dihydralazine, IV ketanserin, IV nicardipine, IV urapidil, and IV diazoxide. In addition, nifedipine may be preferred as the first-line agent. There was no difference in the risk of maternal hypotension, maternal and fetal outcomes, and adverse effects between the drugs, except for adverse effects in IV dihydralazine and IV ketanserin.

## 1. Introduction

Hypertension is the most common cardiovascular disorder during pregnancy, which occurs in 5–10% of pregnancies, and causes poor mortality and morbidity for both mother and child [1,2]. In the early term of pregnancy, the blood pressure (BP) generally drops temporarily as an adaptation process, but then increases as the pregnancy progresses [3]. Hypertensive disorders in pregnancy increase the risks of preterm birth, placental abruption, fetal growth restriction, and other complications [4]. The risk of complication is related to the severity of BP elevation [4]. Complications caused by hypertension during pregnancy need to be taken seriously, especially severe hypertension [5]. Moreover, women with a history of hypertensive disorders in pregnancy have a risk of developing cardiovascular disorders later in life [6,7].

Severe hypertension during pregnancy is defined as a condition of systolic blood pressure (SBP) ≥ 160 or diastolic blood pressure (DBP) ≥ 110 mmHg [8,9]. This is an emergency situation that requires immediate antihypertensive medications to be administered to lower the BP [10,11,12]. Antihypertensive drugs are widely available and have been compared in clinical trials to assess their effectiveness in severe hypertension in pregnancy. Labetalol, hydralazine, or nifedipine are among the most commonly used antihypertensive drugs to manage severe hypertension in pregnancy [5,8,12,13]. Several international guidelines define BP targets in pregnancy differently. The American College of Obstetricians and Gynecologists (ACOG) recommends that antihypertensive medication be administered when the BP is ≥160/110 mmHg, whereas the National Institute for Health and Care Excellence (NICE) recommends antihypertensive medication to be initiated when the BP is ≥140/90 mmHg. On the other hand, the European Society of Cardiology (ESC) recommends that antihypertensives be administered when the BP is ≥150/95 mmHg [1,8,9]. 

The presence of adverse effects, such as a sudden drop in BP, should be avoided [14]. Women with hypertension in pregnancy are also at risk of preeclampsia [15]. Preeclampsia is defined as the new onset of high BP (persistent BP ≥ 140 mmHg systolic and/or ≥90 mmHg diastolic) that occurs after 20 weeks of gestation, accompanied by one of the following complications: new onset of proteinuria, thrombocytopenia, kidney disorders, liver disorders, or pulmonary edema [8]. Preeclampsia may increase the risk of maternal and fetal mortality and morbidity [16]. 

Hypertensive disorders are responsible for 14% of maternal deaths worldwide [17]. Maternal mortality is more likely to occur when the BP ≥ 160/110 mmHg [18]. Based on a retrospective study over 5 years (2011–2016), 57.3% of pregnant women with preeclampsia had severe hypertension called severe preeclampsia [19]. Risk factors for severe preeclampsia include multiple pregnancies, overweight, obesity, nulliparity, and diabetes [20]. Multiple pregnancies (pregnant with twins or triplets) and obesity are the strongest risk factors for severe preeclampsia, whereby women with multiple pregnancies or obesity have a fourfold risk of developing severe preeclampsia [20].

Recommendations to control the BP are one aspect of expectant management, instead of immediate delivery, especially for extremely preterm babies, in women with preeclampsia or severe hypertension in pregnancy [8]. However, delivery is recommended any time if the maternal or fetal condition shows deterioration, such as uncontrolled severe hypertension, stroke, haemolysis, elevated liver enzymes, low platelet count (HELLP) syndrome, etc. [8]. Expectant management is associated with higher gestational age, reduced fetal morbidity, and reduced length of intubation among neonates [21,22]. In addition, it is associated with lower cases of maternal intraventricular hemorrhage or hyaline membrane disease, and less requirement for maternal ventilation [21]. 

Uncontrolled hypertension causes 35.6% of women with severe preeclampsia to deliver the offspring immediately [23]. Premature delivery is common among these women, in addition to an increased risk of undergoing a cesarean section and having babies with low birth weight [2]. Hypertensive disorders in pregnancy and preeclampsia also increase the risk of preterm deaths [24]. Globally, premature birth causes 15% of mortality in infants [25]. Based on data from the World Health Organization (WHO), almost 99% of preterm deaths are caused by complications during pregnancy [26].

Effective treatment for severe hypertension in pregnancy is necessary to protect the mother and child from the risk of complications. Although several antihypertensive agents are used to treat severe hypertension, evidence on their effectiveness and safety profile is inconclusive. Therefore, this systematic review aimed to comprehensively assess the effectiveness and safety of antihypertensive agents for severe hypertension in pregnancy. The parameters used in this systematic review include the risk of persistent severe hypertension, risk of maternal hypotension, adverse effects, and maternal and fetal outcomes. 

## 2. Material and Methods

### 2.1. Search Strategy

The systematic review was conducted following Preferred Reporting Items for Systematic Reviews and Meta-Analyses (PRISMA). A literature search was performed using the databases MEDLINE (PubMed) and Cochrane Library, up until August 2021. Severe hypertension was defined as a condition of SBP ≥ 160 and/or DBP ≥ 110 mmHg [8,9]. Search strategies included the use of the following terms: (“Hypertension, Pregnancy-Induced” [Mesh]) OR (“gestational hypertension” [tw] OR “Preeclampsia” [Mesh]) AND “Antihypertensive Agents” [Mesh]. The MEDLINE (PubMed) and Cochrane searches included only articles reporting randomized controlled trials (RCTs). The PRISMA checklist is provided in the Supplementary materials Table S1.

### 2.2. Study Selection

**Figure 1:**
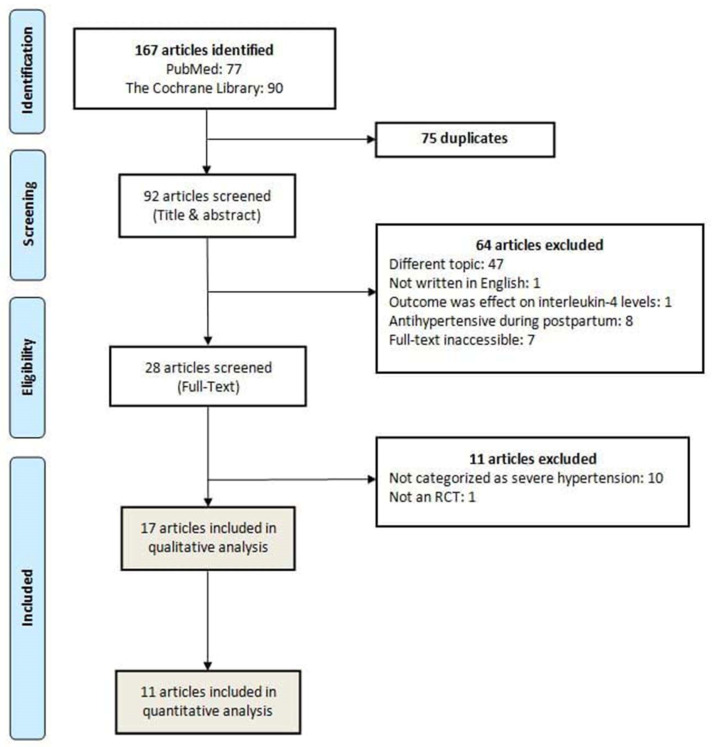
PRISMA flowchart: literature search results.

All search records from an electronic database were exported into the Mendeley Reference Manager and checked for duplicates. Screening was conducted by title and abstract, followed by selection based on a full-text article. The inclusion criteria used in the screening process included RCTs on antihypertensive medications in pregnancy and corresponding to the population, intervention, comparison, and outcome (PICO) analysis provided in Table 1. Articles excluded in the initial screening process were those reporting on studies involving animals and cells, studies conducted in the postpartum period, available in languages other than English, or when the full texts were inaccessible. Observational studies (cross-sectional, retrospective/prospective cohort, case–control designs, and non-intervention arms of RCTs), case series, case reports, and irrelevant studies were also excluded from this review. The PRISMA flowchart template for study selection is depicted in Figure 1.

### 2.3. Data Extraction

Data extraction was performed by manually entering the required information into the predetermined form of data extraction. The data extracted included author, year of publication, country, study design, definition of severe gestational hypertension or preeclampsia, number of participants, intervention arm, BP target, and observation period.

### 2.4. Risk-of-Bias and Quality Assessments

Each study was assessed for the quality and risk-of-bias using the Cochrane risk-of-bias 2 (RoB 2) tool for RCTs [27]. Five aspects were assessed for risk-of-bias, including bias arising from the randomization process, deviations from the intended intervention, missing outcome data, measurement of the outcome, and selection of the reported result [28]. The overall bias from each study was classified based on RoB 2 guidelines as high risk, some concerns, or low risk-of-bias [27].

### 2.5. Meta-Analysis

Cochrane Review Manager 5.4 (https://training.cochrane.org/online-learning/core-software-cochrane-reviews/revman/ (accessed on 6 September 2021)) was used to perform statistical analyses for the meta-analysis. The analysis was attached in subgroups based on a comparison of antihypertensive medication. Meta-analysis was performed with a random-effect model to summarize all outcomes from the studies included. The effect size was presented as a relative risk (RR) with a 95% confidence interval (CI). For studies with significant RR, the risk difference (RD) was also calculated. Heterogeneity was analyzed using τ^2^, chi^2^, and I^2^.

## 3. Results

### 3.1. Study Characteristics

The initial search identified 167 articles in both databases. After removing 75 duplicates, 92 articles were screened by title and abstract, in which 64 articles were further excluded. In the full-text screening of 28 articles, 17 RCT articles met the inclusion criteria, consisting of 2312 women as participants [5,12,13,29,30,31,32,33,34,35,36,37,38,39,40,41,42]. In three studies, 41 women were excluded for the following reasons: nine women decided to discontinue the intervention in one study [5]; 27 women were postnatal patients in one study [41]; and in one study, two were postpartum mothers, one woman was terminated due to early delivery before intervention was administered, one woman was incorrectly identified, and one woman was randomized twice [28]. One study with the most number of participants (894 women) compared interventions with three different antihypertensive agents: nifedipine, labetalol, and methyldopa [12]. Overall, the majority of women received the following medications: nifedipine (33.51%), labetalol (31.48%), methyldopa (13.25%), and hydralazine (10.83%). Other antihypertensive medications were prazosin, dihydralazine, diazoxide, ketanserin, nicardipine, and urapidil [30,31,37,38,39,41]. In addition, only 11 studies were included in the quantitative analyses because the other six studies presented different and incomparable outcome measures related to our inclusion criteria.

Each drug was administrated in various dosage forms, as seen in Table 2. There were nine different medication comparisons from all the studies, in which seven studies (580 women) compared nifedipine with labetalol [13,32,33,34,35,36,42], two studies (195 women) compared nifedipine with hydralazine [5,29], two studies (74 women) compared ketanserin with dihydralazine [30,31], one study (200 women) compared hydralazine with labetalol [40], one study (145 women) compared nifedipine with prazosin [38], one study (97 women) compared diazoxide with hydralazine [41], one study (60 women) compared nicardipine with labetalol [37], one study (26 women) compared urapidil with dihydralazine [39], and one study (894 women) compared three intervention arms: nifedipine, labetalol, and methyldopa [12]. Varying doses and durations of drug therapy were observed in each study. 

The vast majority of studies included defined severe hypertension as SBP ≥ 160 mmHg and DBP ≥ 110 mmHg, but two studies used different definitions, whereby severe hypertension was defined as SBP ≥ 170 mmHg and DBP ≥ 110 mmHg [37,41]. Almost all studies defined the following BP targets of SBP ≤ 150 mmHg and DBP ≤ 100 mmHg. However, some studies had different BP targets, including three studies with SBP ≤ 140 mmHg and DBP ≤ 100 mHg [33,34,41], one study with a BP target of ≤160/110 mmHg [40], and one study with a BP target of a decrease of 20% arterial BP [37]. The characteristics of studies included are provided in Table 3, summary of results from the meta-analysis is provided in Table 4 and summary of interventions effects is provided in Table 5.

### 3.2. Risk-of-Bias Assessment

**Figure 2:**
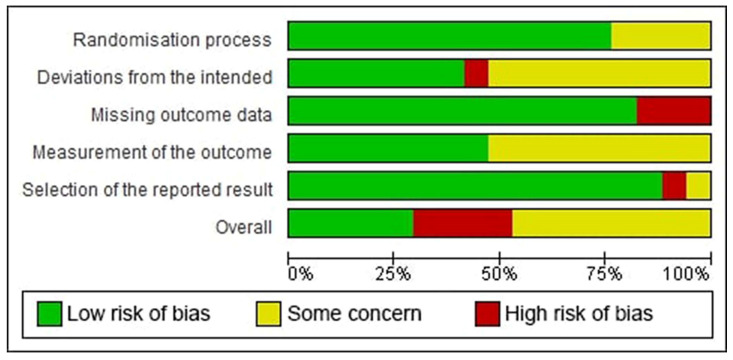
Risk-of-bias graph: the authors’ judgments for each risk-of-bias item in the studies included, provided in percentage form.

**Figure 3:**
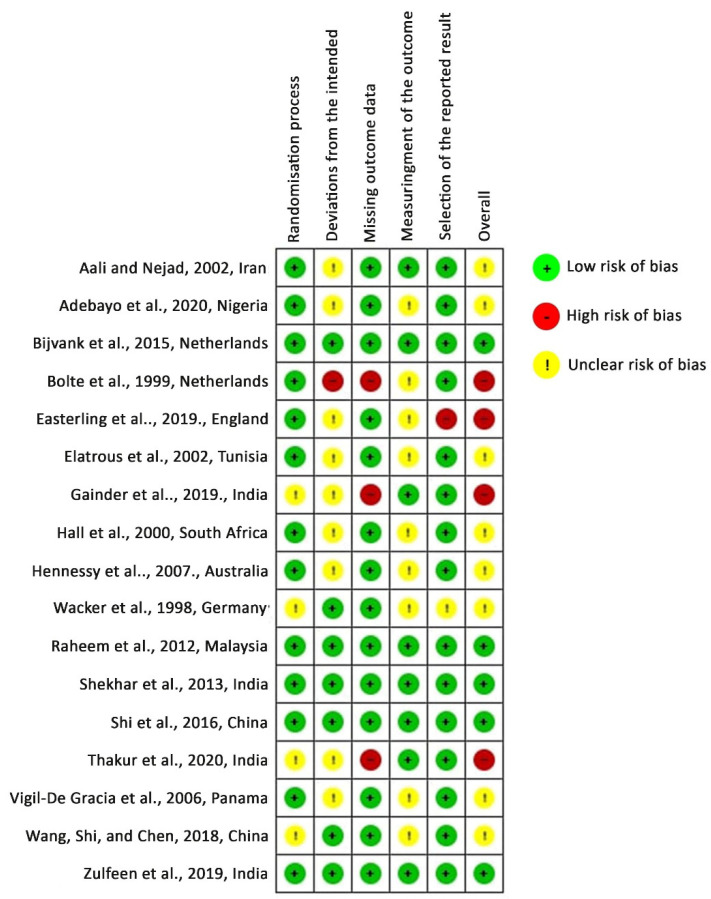
Risk-of-bias assessment: authors’ judgments for risk-of-bias items in each study included. Studies are listed alphabetically by author name.

According to the RoB 2 tool, five studies were identified as having low risk-of-bias, eight studies had some concern for risk-of-bias, and four studies had a high risk-of-bias. In detail, 23.53% had some concern for the randomization process and 52.94% had some concern at deviations from the intended interventions. A high risk-of-bias arose from deviations from the intended interventions in one study [30], from missing outcomes in three studies [30,33,34], and from selection bias in one study [12]. Figure 2 and Figure 3 depict the risk-of-bias assessments of the studies included.

### 3.3. Effect of Interventions

#### 3.3.1. Nifedipine versus Labetalol

**Figure 4:**
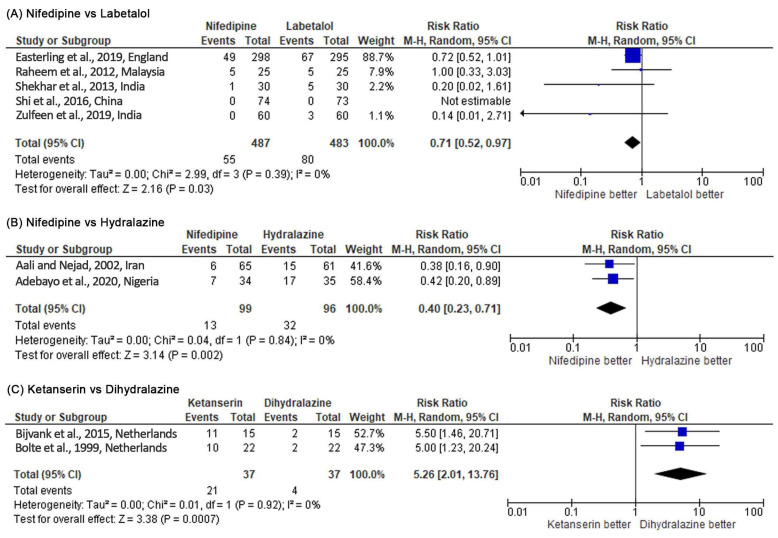
Meta-analysis outcome 1: comparison of antihypertensive medication effectiveness in controlling blood pressure assessed by the risk of persistent severe hypertension incidence. The risk ratio (RR) was used to interpret the risk of persistent severe hypertension incidence after administering the drug. *p*-value in the overall effect shows the significance of the RR.

**Figure 5:**
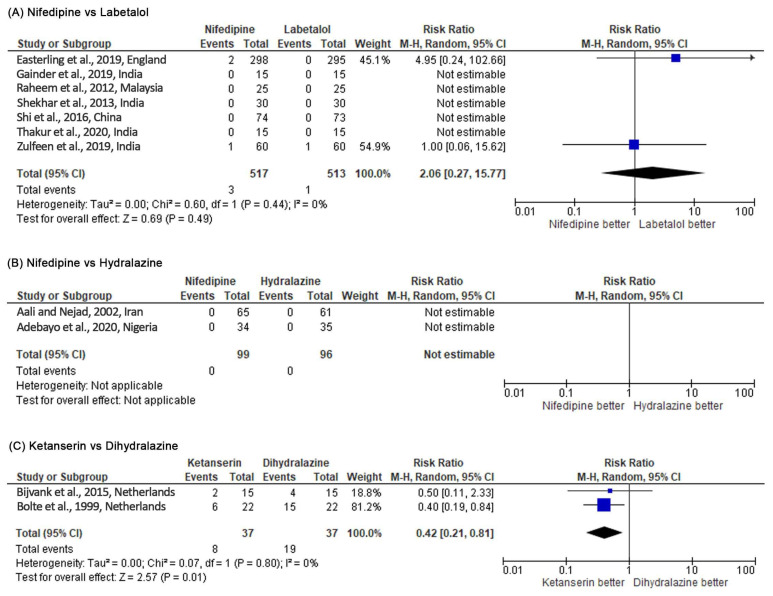
Meta-analysis outcome 2: antihypertensive medication safety comparison assessed by the risk of maternal hypotension incidence. The risk ratio (RR) was used to interpret the risk of maternal hypotension incidence after administering the drug. *p*-value in the overall effect shows the significance of the RR.

Nifedipine had a significantly lower risk in persistent high BP than labetalol (RR 0.71, 95% CI 0.52–0.97, *p* = 0.03; five studies) (see Figure 4). However, when the RD was used as a summary statistic (RD –0.04, 95% CI –0.11 to 0.02; five studies), heterogeneity between the studies rose to 76% and sensitivity analysis became insignificant (*p* = 0.19). There was no significant difference between the two groups for maternal hypotension (RR 2.06, 95% CI 0.27–15.77, *p* = 0.49; seven studies). However, more hypotension occurred in the nifedipine group (see Figure 5). There was no significant difference for the incidence of adverse effects, but nifedipine was associated with more adverse effects. Similarly, there was no significant difference in maternal and fetal outcomes, including maternal death, neonatal death, eclampsia, pulmonary edema, renal failure, placental abruption, cesarean delivery, stillbirth, abnormal fetal heart rate, and the Appearance, Pulse, Grimace, Activity, Respiration (APGAR) score. 

#### 3.3.2. Nicardipine versus Labetalol

Only one study compared nicardipine with labetalol. No significant difference was found in the nicardipine and labetalol groups for persistent high BP (RR 0.82, 95% CI 0.40–1.68, *p* = 0.59) and adverse effects (RR 1.07, 95% CI 0.76–1.57, *p* = 0.70). There was no incidence of maternal hypotension between the two groups.

#### 3.3.3. Nifedipine versus Hydralazine

Nifedipine was more effective in controlling BP than hydralazine (RR 0.40, 95% CI 0.23–0.71, *p* = 0.002; two studies) (see Figure 4). There was no incidence of hypotension and maternal mortality in both study arms (two studies) (see Figure 5). There was no significant difference for the incidence of adverse effects, but nifedipine had a higher risk of headache. On the other hand, no significant difference was observed for maternal and fetal outcomes (cesarean delivery, perinatal death, stillbirth, neonatal intensive care unit (NICU) admission, and APGAR score) in both study arms. 

#### 3.3.4. Nifedipine versus Prazosin

Only one study compared nifedipine with prazosin. No significant difference was found between nifedipine and prazosin in controlling BP (RR 0.32, 95% CI 0.03–3.00, *p* = 0.32). Similarly, there was no significant difference in maternal and fetal outcomes (eclampsia, kidney disorders, HELLP syndrome, placenta abruption, pulmonary edema, maternal death, miscarriage, and NICU admission) between nifedipine and prazosin.

#### 3.3.5. Ketanserin versus Dihydralazine

Dihydralazine was able to control high BP significantly better than ketanserin (RR 5.26, 95% CI 2.01–13.76, *p* = 0.0007; two studies) (see Figure 4). Based on the RR, dihydralazine was significantly associated with more risk of maternal hypotension (RR 0.42, 95% CI 0.21–0.81, *p* = 0.01) (see Figure 5). However, when the RD was used as a summary statistic, heterogeneity rose to 49% and sensitivity analysis became insignificant (RD –0.27, 95% CI –0.55 to 0.00, *p* = 0.05). Dihydralazine was significantly associated with more adverse effects than ketanserin (RR 0.38, 95% CI 0.23–0.64, *p* = 0.0002; two studies). There was no significant difference in maternal and fetal outcomes (eclampsia, HELLP syndrome, placenta abruption, cesarean delivery, and maternal and neonatal mortality) between these drugs.

#### 3.3.6. Urapidil versus Dihydralazine

Only one study compared urapidil with dihydralazine. The outcomes of the study included adverse effects, eclampsia, cesarean delivery, abnormal fetal heart rate, and neonatal death. No significant difference was reported in maternal outcomes and neonatal deaths in both groups, and there was no incidence of adverse effects.

#### 3.3.7. Hydralazine versus Labetalol

A comparison of effectiveness between hydralazine and labetalol was conducted in one study. The results indicated no difference in efficacy for controlling BP between the two groups (RR 1.00, 95% CI 0.30–3.35, *p* = 1.00) and no significant difference in the adverse effect incidence between hydralazine and labetalol. Adverse effects, such as palpitations, were significantly more common in the hydralazine group (RR 5.00, 95% CI 1.12–22.24, *p* = 0.03). There was no significant difference for maternal hypotension in both groups (RR 5.00; 95% CI 0.42–102.85, *p* = 0.30). In addition, no significant difference was noted in maternal and neonatal outcomes (eclampsia, pulmonary edema, cesarean delivery, neonatal hypotension, neonatal complications, abnormal fetal heart rate, NICU admission, HELLP Syndrome, and APGAR score).

#### 3.3.8. Hydralazine versus Diazoxide

Only one study compared the effectiveness of hydralazine with diazoxide. There was no difference in the risk of persistent high BP between the two groups (RR 1.06, 95% CI 0.55–2.05, *p* = 0.85). Similarly, there was no significant difference between both groups in maternal and fetal outcomes (cesarean birth, APGAR score, hypoglycemia and neonatal respiratory distress syndrome, and perinatal death). 

#### 3.3.9. Methyldopa versus Nifedipine

A comparison of the effectiveness between methyldopa and nifedipine was reported by only one study. Nifedipine use was shown to be associated with a lower risk of persistent high BP (RR 1.43, 95% CI 1.03–1.99, *p* = 0.03). The risk of maternal hypotension was not significantly different between the two groups (RR 0.20, 95% CI 0.01–4.11, *p* = 0.30). However, nifedipine was associated with more adverse effects than methyldopa (RR 0.56, 95% CI 0.42–0.75, *p* = 0.0001). In addition, more infants in the nifedipine group were admitted to the NICU (RR 0.54, 95% CI 0.36–0.83, *p* = 0.005). There were no maternal deaths and no significant differences in adverse effects and maternal and fetal outcomes (placental abruption, cesarean delivery, neonatal death, stillbirth, abnormal fetal heart rate, and APGAR score) between these drugs.

#### 3.3.10. Methyldopa versus Labetalol

Only one study compared the effectiveness of methyldopa and labetalol. The study found no significant difference between the two groups in controlling the BP (RR 1.04, 95% CI 0.78–1.39, *p* = 0.80). None of the mothers in either group experienced hypotension or death. Other maternal and fetal outcomes (placental abruption, cesarean delivery, neonatal death, stillbirth, abnormal fetal heart rate, NICU admission, and APGAR score) and adverse effects were found to be nonsignificant n both groups.

## 4. Discussion

The effectiveness of antihypertensives was assessed by the risk of persistent severe hypertension, whereas drug safety was assessed by maternal hypotension, maternal and fetal outcomes, and adverse effects. The outcomes between studies were compared in a meta-analysis. Of 17 studies included, we could not include six studies in the meta-analysis because the outcome measure data compatible with our inclusion criteria were not considered in those studies. Most comparative drug studies of severe hypertension treatment during pregnancy come from middle-income countries, although data were still sparse [11,44]. The definitive recommendation in these limited-resource countries is prominent because prioritization in health intervention, including BP-lowering drugs, can significantly reduce the health burden related to hypertension in pregnancy. Therefore, the use of effective antihypertensive agents to control BP was crucial [45].

The results of our meta-analysis estimated that oral nifedipine can significantly lower the risk of persistent high BP in pregnancy compared to intravenous (IV) hydralazine and IV labetalol, with no differences in the incidences of maternal hypotension and adverse effects, and on maternal and fetal outcomes. One of the studies comparing nifedipine and labetalol had a dominating number of participants that caused the study weight to become disproportionate [12]. The significance of RR in persistent high BP between nifedipine and labetalol should be viewed with caution because sensitivity and heterogeneity in the analysis become insignificant when the RD was used as a summary statistic. However, the therapeutic success rate favored oral nifedipine over IV hydralazine and IV labetalol. There was a significant difference in beneficial effects between oral nifedipine and IV hydralazine but not for IV labetalol. It was concluded that oral nifedipine was as efficacious as IV labetalol. Our study also showed that IV dihydralazine was significantly more effective than IV ketanserin in controlling BP during pregnancy. The results of our meta-analysis show that IV dihydralazine had more adverse effects than IV ketanserin. However, the use of IV dihydralazine did not show any difference in the risk of maternal hypotension, or maternal and fetal outcomes. 

Several previous studies confirm our present findings. Duley et al. reported that nifedipine was significantly more effective in lowering the risk of persistent high BP during pregnancy than hydralazine, and that there was no difference between nifedipine, labetalol, and hydralazine in the risk of maternal hypotension, adverse effects, and on maternal and fetal outcomes [11]. Duley et al. also reported that IV dihydralazine was more effective in lowering the risk of persistent high BP than IV ketanserin. With regard to other outcomes, such as maternal hypotension, their results showed no difference in the risk of maternal hypotension between the two interventions (IV dihydralazine and IV ketanserin), but IV dihydralazine had more adverse effects than IV ketanserin, which is consistent with the results of our meta-analysis. Duley et al. used the random-effect model only if the heterogeneity was substantial (I^2^ > 30%, T^2^ > 0, or *p* < 0.1). Their study used the previous version of the RoB tool to assess the quality of studies; however, in our systematic review, we used the recent RoB tool (RoB 2.0). In addition, our systematic review contains recent studies with larger sample sizes and one of them was a multicenter study [12].

Another systematic review was conducted by Alavifard et al. using a network meta-analysis [44]. Similar to our findings, Alavifard et al. reported that oral nifedipine provides the highest therapeutic success rate for controlling BP in pregnancy compared to IV labetalol and IV hydralazine. A significant difference in effectiveness was observed when oral nifedipine was compared with IV hydralazine, but not with IV labetalol. Alavifard et al. also reported that there was no difference in the risk of adverse effects between oral nifedipine, IV labetalol, and IV hydralazine. Unfortunately, the risk of maternal hypotension was not assessed in Alavifard et al.’s study because of low event rates; therefore, meta-analysis for maternal hypotension could not be compared with our findings. However, a higher frequency of hypotension occurred with hydralazine use (hydralazine 7.6%, labetalol 1.7%, and nifedipine 0.6%) [44]. Regarding the number of participants in the effectiveness comparison between nifedipine and labetalol, Alavifard et al. had a smaller number of participants (490) than our study (970), which might cause differences in the size of the CI.

There were different findings from network meta-analysis by Sridharan et al. [46]. Their analysis showed no difference in effectiveness in controlling the BP in pregnancy for nifedipine compared to hydralazine (OR 2.1, 95% CI 0.9–5.2) and labetalol (OR 0.7, 95% CI 0.3–1.5). The wide estimate interval in the results of network meta-analysis caused unclear reliability. Sridharan et al. reported that adequate evidence was used to compare nifedipine and hydralazine against labetalol [46]. However, sufficient evidence regarding the comparison between nifedipine and hydralazine in pregnant women was still sparse; therefore, the recommendation was based on limited data.

This systematic review consists of recent studies and various intervention arms; therefore, the use of antihypertensive medication was more widely described. A secondary analysis was used in the meta-analysis using the RD to determine any significant effect of the RR. This systematic review was also based on PRISMA guidelines and the quality of the studies was assessed based on the recent Cochrane RoB 2 tool. 

Inevitably, there were some limitations in this systematic review. First, only limited information was retrieved from some studies; hence, our meta-analysis had to exclude those studies. Second, this review included studies of various qualities. Finally, there were variations in the definition of outcome measures used in the studies included (e.g., severe hypertension and BP target). Therefore, we use broader inclusion criteria to standardize the different characteristics of each study. Despite these variations, most importantly, a clear definition was used for severe hypertension (SBP ≥ 160 mmHg or DBP ≥ 110 mmHg) and the studies included reported at least one outcome of the effectiveness or safety of antihypertensives. From the results of this study, we recommend that oral nifedipine be the first-choice drug in daily clinical practice as therapy for severe hypertension during pregnancy. For further research, a comprehensive economic evaluation related to preeclampsia therapy is also needed to generate more conclusive recommendation, due to current limited data about this topic [47].

## 5. Conclusions

Oral nifedipine may be considered the first-line antihypertensive agent for severe hypertension in pregnancy. The results of this review suggested that oral nifedipine had the highest therapeutic success rate in controlling hypertension during pregnancy compared to other common medications used (IV hydralazine and IV labetalol). Oral nifedipine was relatively safe and showed no difference in the risk of hypotension, maternal and fetal outcomes, and incidence of adverse effects compared to IV hydralazine and IV labetalol. However, there was not enough evidence that oral nifedipine was preferable to other antihypertensive medications for severe hypertension in pregnancy. IV dihydralazine could control high BP significantly better than IV ketanserin, with no difference in the risk of hypotension. However, IV dihydralazine was associated with more adverse effects than IV ketanserin. Adequately powered RCTs in the comparison between oral nifedipine and IV labetalol, or other agents such as diazoxide, urapidil, prazosin, and methyldopa, in the treatment of severe hypertension in pregnancy are required to guide a better clinical judgment on the most effective and safe drug. This is especially important in the setting of low- and middle-income countries, where access and drug of choice may be limited. Therefore, a conclusive recommendation can be crucial in the treatment of severe hypertension in pregnancy in clinical practice.

## Figures and Tables

**Table 1:** PICO analysis.

	Main Concept	Synonyms/Abbreviations/More Specific Concepts
P—Problem, condition, patient, population, setting	Pregnant women with severe gestational hypertension or preeclampsia with systolic blood pressure (BP) ≥ 160 mmHg and/or diastolic BP ≥ 110 mmHg	Pregnancy Gestational hypertension Preeclampsia Severe preeclampsia Pregnant women
I—Intervention: dose, delivery, frequency	Antihypertensive medications	Antihypertensive agents Calcium channel blockers
C—Comparison (may not be one)	Not available	Not available
O—Outcome: what happens to P? Cost-effective? Patients’ experience?	Primary: lowered BP Secondary: maternal and perinatal outcomes	Lowered BP Preeclampsia complications Placenta abruption Maternal adverse events Perinatal adverse events Stillbirth Neonatal death

**Table 2:** Dosage forms of drugs administered.

No.	Drugs	Dosage Forms
1.	Labetalol	Intravenous [13,32,33,35,36,37,40,42] Oral [12,34]
2.	Nifedipine	Oral [5,12,13,32,33,34,35,36,38,42] Subglingual [29]
3.	Hydralazine	Intravenous [5,29,40,41]
4.	Dihydralazine	Intravenous [30,31,39]
5.	Ketanserin	Intravenous [30,31]
6.	Nicardipine	Intravenous [37]
7.	Prazosin	Oral [38]
8.	Urapidil	Intravenous [39]
9.	Diazoxide	Intravenous [41]
10.	Methyldopa	Oral [12]

**Table 3:** Characteristics of studies included.

Author, Year of Publication, Country	Methods and Design	Definition of Hypertension or Preeclampsia	Number of Participants	Drug Comparisons			BP Target (mmHg)	Observation Period
Shi et al. [42], 2016, China	Method: -Double-blinded -Block randomized Design: -Gestational age > 30 weeks -Intervention administered every 15 min	Severe preeclampsia BP ≥ 160/110 mmHg; preeclampsia criteria not defined	147	Oral nifedipine	IV Labetalol		≤150/100	1 h
Zulfeen et al. [13], 2019, India	Method: -Double-blinded -Block randomized -Computer generated -Parallel treatments Design: -Gestational age > 28 weeks -Two envelope packages (A&B) A: intervention B: opposite intervention (additional if needed)	Severe hypertension (acute) Sustained BP ≥ 160/110 mmHg on two occasions (30 min apart)	120	Oral nifedipine	IV Labetalol		≤150/100	1 h
Shekhar et al. [35], 2013, India	Method: -Double-blinded -Computer generated -Parallel treatments Design: -Gestational age ≥ 24 weeks -Two envelope packages (A&B) A: intervention B: opposite intervention (additional if needed)	Hypertensive emergency Sustained BP ≥ 160/110 mmHg on two occasions (30 min apart)	60	Oral nifedipine	IV Labetalol		≤150/100	80 min
Raheem et al. [32], 2012, Malaysia	Method: -Double-blinded -Block randomized -Computer generated Design: -Gestational age ≥ 24 weeks -Two envelope packages (A&B) A: intervention B: opposite intervention (additional if needed)	Hypertensive emergency Sustained BP ≥ 160/110 mmHg measured ≥2 occasions in the last 4 h	50	Oral nifedipine	IV Labetalol		≤150/100	1 h
Gainder et al. [33], 2019, India	Method: -Table random numbers Design: -Gestational age 26–40 weeks	Severe hypertension (acute) Sustained BP ≥ 160/105 mmHg	30	Oral nifedipine	IV Labetalol		≤140/90	30 min
Wang, Shi, and Chen [36], 2018, China	Method: -Double-blinded Design: -Intervention administered every 20 min	Severe preeclampsia <34 weeks, BP ≥ 160/110 mmHg, proteinuria ≥ 5 g/24 h	143	Oral nifedipine	IV Labetalol		SBP 130–150/ DBP 80–105	80 min
Easterling et al. [12], 2019, England	Method: -Open label -Multicenter -Parallel treatments Design: -Gestational age ≥ 28 weeks -Additional doses for nifedipine or labetalol treatments if BP > 155/105 mmHg in 1 h	Severe hypertension Sustained BP ≥ 160/110 mmHg on two occasions (15 min apart)	894	Oral nifedipine	Oral labetalol	Oral methyldopa	SBP 120–150/ DBP 70–100	Nifedipine and labetalol: 2 h Methyldopa: 6 h
Thakur et al. [34], 2020, India	Method: -Table random numbers Design: -Gestational age 26–40 weeks	Severe hypertension (acute) Sustained BP ≥ 160/110 mmHg	30	Oral nifedipine	IV Labetalol		≤140/90	30 min
Elatrous et al. [37], 2002, Tunisia	Method: -Single-blinded Design: -Gestational age ≥ 24 weeks	Severe hypertension Sustained BP ≥ 170/110 mmHg on two occasions (30 min apart)	60	IV Nicardipine	IV Labetalol		Achieved sustained 20% decreased in arterial BP	1 h
Aali and Nejad [29], 2002, Iran	Method: -Single-blinded -Block randomized Design: -Gestational age > 20 weeks -Intervention repeated when BP target was not achieved after 20 min	Severe preeclampsia BP ≥ 160/110 mmHg, proteinuria ≥ 5 g/24 h	126	Sublingual nifedipine	IV Hydralazine		DBP 90–100	20 min
Adebayo et al. [5], 2020, Nigeria	Method: -Parallel -Open label -Online randomization service -Single block randomization Design: -Gestational age ≥ 28 weeks -Intervention repeated every 30 min until BP target achieved	Severe hypertension Sustained BP ≥ 160/110 mmHg on two occasions (30 min apart)	78	Oral nifedipine	IV Hydralazine		SBP 140–150/ DBP 90–100	2 h
Hall et al. [38], 2000, South Africa	Method: -Open label Design: -Cannot controlled by methyldopa 2 g/day -Crossover when BP target not achieved	Severe hypertension and preeclampsia (early-onset) Referring to Davey and MacGillivray [43]	150	Oral nifedipine	Oral prazosin		<160/110	24 h
Bijvank et al. [31], 2015, Netherlands	Method: -Double-blinded - Block randomization Design: -Gestational age ≤ 32 weeks - Double dummy: -Methyldopa 200 mg/day as standard co-medication	Severe hypertension and preeclampsia (early-onset) Pregnant women with DBP ≥ 110 mmHg and proteinuria ≥ 300 mg/24 h	30	IV Ketanserin	IV Dihydralazine		DAP = 85–100	2 h
Bolte et al. [30], 1999, Netherlands	Method: -Open label -Multicenter -Block randomized -Parallel Design: -Gestational age 26–32 weeks -Conducted in four hospitals	Severe preeclampsia (early-onset) DP > 110 mmHg or elevation DBP ≥ 20 mmHg compared with 20 weeks’ gestational DBP in chronic hypertension	44	IV Ketanserin	IV Dihydralazine		70–95 (intra-arterial) or 85–110 (sphygmomanometer)	3 h
Wacker et al. [39], 1998, Germany	Method: -Computer-generated randomization -Not blinded -Parallel Design: -Gestational age 26–38 weeks -Both treatments received MgSO_4_	Hypertension and preeclampsia Sustained BP ≥ 160/110 mmHg on two occasions 3 h apart with complete bed rest One or more: proteinuria, generalized, edema, hyperflexia	26	IV Urapidil	IV Dihydralazine		DBP 90–95	6 h
Vigil-De Gracia et al. [40], 2006, Panama	Method: -Not blinded -Computer-generated randomization Design: -Gestational age ≥ 24 weeks -Intervention administered every 20 min	Severe hypertension BP ≥ 160/110 mmHg	200	IV Hydralazine	IV Labetalol		<160/110	80 min
Hennessy et al. [41], 2007, Australia	Method: -Randomized by sequential selection of numbers Design: -BP recorded 20 and 60 min post drug administration	Hypertensive emergency BP > 170/110 mmHg or >140/90 mmHg with preeclampsia symptoms; preeclampsia criteria not defined	124	IV Diazoxide	IV Hydralazine		≤140/90	1 h

Abbreviations: BP, blood pressure; mmHg, millimeters of mercury (hydrargyrum); IV, intravenous; SBP, systolic blood pressure; DBP, diastolic blood pressure; DAP, diastolic arterial pressure.

**Table 4:** Summary of meta-analysis comparing antihypertensive agents.

Outcome or Subgroup	Number of Studies	Participants	Statistical Method	RR [95% Cl; *p*-Value]
Persistent high blood pressure				
Nifedipine vs. labetalol	5	970	RR (M-H, random, 95% CI)	0.71 [0.52, 0.97; 0.03]
Nifedipine vs. hydralazine	2	195	RR (M-H, random, 95% CI)	0.40 [0.23, 0.71; 0.002]
Ketanserin vs. dihydralazine	2	74	RR (M-H, random, 95% CI)	5.26 [2.01, 13.76; 0.0007]
Maternal hypotension				
Nifedipine vs. labetalol	7	1030	RR (M-H, random, 95% CI)	2.06 [0.27, 15.77; 0.49]
Nifedipine vs. hydralazine	2	195	RR (M-H, random, 95% CI)	Not available
Ketanserin vs. dihydralazine	2	74	RR (M-H, random, 95% CI)	0.42 [0.21, 0.81; 0.01]

Abbreviations: RR, risk ratio; CI, confidence interval; M-H, mantel haenszel. RR = 1 means no difference effect between the two interventions; RR > 1 means intervention 1 (left side) has a greater risk of persistent high BP or maternal hypotension than intervention 2 (right side); RR < 1 means intervention 1 has a protective effect on persistent high BP or maternal hypotension compared to intervention 2.

**Table 5:** Summary of intervention effects.

Drug Comparisons		Results		
		Persistent High BP	Maternal Hypotension	Other Side Effects
Nifedipine	Labetalol	Nifedipine had a lower risk (RR 0.71, p = 0.03; five studies)	No significant difference (RR 2.06, p = 0.49; seven studies)	No significant difference (participant experience) (RR 0.75, p = 0.28; two studies) Nifedipine: at risk of having more side effects (RR 1.57, p = 0.02; six studies)
Nicardipine	Labetalol	No significant difference (RR 0.82, p = 0.59; one study)	No incident (one study)	No significant difference (total side effects) (RR 1.07, p = 0.70; one study)
Nifedipine	Hydralazine	Nifedipine had a significantly lower risk (RR 0.40, p = 0.002; two studies)	No incident (two studies)	No significant difference (participant experience) (RR 1.03, p = 0.94; one study) Nifedipine: more at risk of headache (RR 3.69, p = 0.02; two studies)
Nifedipine	Prazosine	No significant difference (RR 0.32, p = 0.32; one study)	Not reported	Not reported
Ketanserine	Dyhidralazine	Dihydralazine had a lower risk (RR 5.26, p = 0.0007; two studies)	Dihydralazine more at risk (RR 0.42, p = 0.01; two studies)	Dihydralazine: at risk of having more side effects (RR 0.38, p = 0.0002; two studies)
Urapidil	Dyhidralazine	Not reported	Not reported	No incidence (one study)
Hydralazine	Labetalol	No significant difference (RR 1.00, p = 1.00; one study)	No significant difference (RR 5.00, p = 0.30; one study)	No significant difference (participant experience) (RR 1.06, p = 0.86; one study) Hydralazine: more at risk of palpitasion (RR 5.00, p = 0.03; one study)
Hydralazine	Diazoxide	No significant difference (RR 1.06, p = 0.85; one study)	Not reported	Not reported
Methyldopa	Nifedipine	Methyldopa had a lower risk (RR 1.43, p = 0.03; one study)	No significant difference (RR 0.20, p = 0.30; one study)	Nifedipine: at risk of having more side effects (RR 0.56, p = 0.0001; one study)
Methyldopa	Labetalol	No significant difference (RR 1.04, p = 0.8; one study)	No incident (one study)	No significant difference (total side effects) (RR 1.20, p = 0.32; one study)

Abbreviations: BP, blood pressure; RR, risk ratio. Calcium Channel Blocker versus Labetalol.

## Data Availability

The data presented in this study are available in the manuscript and supplementary material.

## Supplementary Materials

The following supporting information can be downloaded at: https://www.mdpi.com/article/10.3390/healthcare10020325/s1, Table S1: PRISMA 2020 main checklist.
